# Feel it to learn it!—Cognitive and motivational effects of haptic learning materials

**DOI:** 10.3389/fpsyg.2026.1753336

**Published:** 2026-03-09

**Authors:** Hannah Sophie Erdmann, Michael Montag, Lena Büscher, Steffi Zander

**Affiliations:** Faculty of Social and Behavioural Sciences, Friedrich Schiller University Jena, Jena, Germany

**Keywords:** anatomical models, cognitive load, haptic learning, intrinsic motivation, learning with models, Need for Touch

## Abstract

**Introduction:**

Haptic learning materials have been increasingly recognized for their potential to enhance cognitive and motivational aspects of learning. This study examines the effects of using haptic anatomical models compared to visual representations in learning environments.

**Methods:**

The research specifically investigates the impact of haptic materials on learning success, intrinsic motivation, and cognitive load, considering individual differences in the Need for Touch (NFT). Using an experimental design, 87 university students participated in a study where they engaged with either haptic or visual materials on anatomical structures.

**Results:**

Results showed no significant difference in overall learning success between the two conditions. However, haptic materials led to lower extraneous cognitive load and higher germane cognitive load in certain contexts, suggesting improved information processing. Additionally, intrinsic motivation was significantly higher in the haptic condition for specific learning content. Regression analyses revealed that students with a high NFT particularly benefited from haptic models, experiencing reduced extraneous load and increased intrinsic motivation.

**Discussion:**

These findings highlight the relevance of haptic materials in optimizing cognitive load and motivation, contributing to embodied learning theories. Future research should explore the long-term effects of haptic learning and its applications across diverse educational contexts.

## Introduction

Learning with models and materials is a long-standing instructional approach and remains widely used in education. From a contemporary perspective, such materials can be understood as one form of multiple external representations (MERs), i.e., different representations (e.g., text, images, formulas, or physical objects) that are provided to support understanding and problem solving. Research on MERs suggests that learning benefits particularly when representations complement each other and help constrain misinterpretations (Ainsworth, 2006). Haptic models (e.g., anatomical models of the heart, brain, or molecules) represent an external representation that, unlike purely visual materials, enables tactile interaction with the learning object. On a sensory and cognitive level, this means that the learning object is examined using exploration procedures that provide additional information about properties such as temperature, spatial extent, sub-processes, texture and material density (Klatzky and Lederman, 2002). Haptics can support learning by making it easier to establish a connection between instructions and physical requirements (Minogue and Jones, 2006).

In the area of learning with media, the form of representation plays a decisive role, which can be explained by several theories and models.

The *Cognitive-Affective Theory of Learning with Media* (CATLM; Moreno and Mayer, 2007) extends the *Cognitive Theory of Multimedia Learning* (CTML; Mayer, 2005) by incorporating affective and metacognitive components. It posits that information is processed through separate channels with limited capacity (Sweller, 1999) and that meaningful learning requires active cognitive processes such as selecting, organizing, and integrating new information (Mayer and Moreno, 2003). Motivation influences cognitive effort (Pintrich, 2003), metacognitive processes regulate emotions and learning strategies (McGuinness, 1990), and prior knowledge shapes learning success (Kalyuga et al., 2003; Moreno, 2004; Moreno and Durán, 2004).

Recent studies have extended the CATLM framework to embodied and haptic learning contexts. Evidence shows that tactile and immersive interactions can reduce extraneous cognitive load and support motivation and engagement. For example, haptic augmented reality applications have been found to lower cognitive load while maintaining intrinsic motivation (Fehrmann, 2025), and AR-based learning objects can enhance learning outcomes by optimizing cognitive resources (Michel-Acosta et al., 2024). Conceptually, the notion of *non-cognitive load* integrates emotional and motivational demands into cognitive load theory (Sultanova, 2025), while research on embodied cognition shows that physical interaction can improve learning without increasing cognitive strain (Ratcliffe and Tokarchuk, 2020).

Together, these findings reinforce CATLM's view that cognition, emotion, and motivation interact dynamically during multimedia learning and highlight the added value of embodied and sensory engagement in (digital) learning environments enriched by haptic MERs.

**Figure 1:**
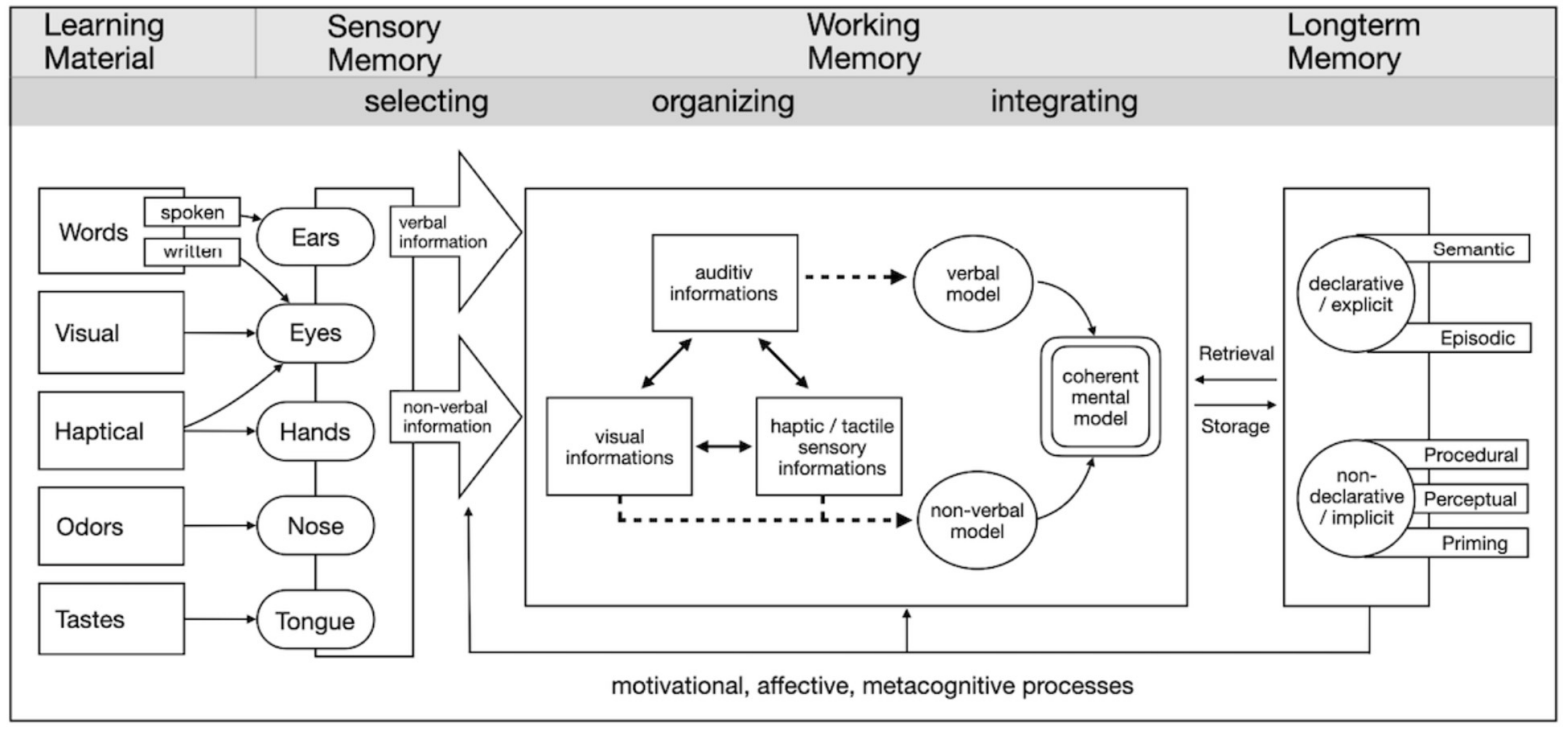
Multisensory model of cognitive—effective learning processing (extended according to Moreno, 2006).

As shown in Figure 1, learning material initially enters the sensory memory via different sensory channels. Words (spoken or written) are received via the auditory or visual system, while visual, haptic, olfactory and gustatory stimuli are processed via the eyes, hands, nose and tongue. The sensory information is then selected, depending on its relevance, and fed into the working memory. In the working memory, this information is processed further and converted into different representations: Auditory information can be converted into a verbal model, while visual and haptic-tactile information is predominantly incorporated into a non-verbal model. These models are merged into a coherent mental model through active cognitive processes (e.g., organization and integration). Motivational, affective and metacognitive processes have a significant influence on this process, for example by controlling attention, regulating cognitive effort or supporting the selection of suitable learning strategies. While haptic exploration provides additional sensory information such as texture or density—which also activates motor and somatosensory areas of the brain—the visual-verbal pathway requires a more symbolic representation of knowledge. Both pathways can ultimately contribute to the formation of coherent mental models that are integrated with existing knowledge in long-term memory and stored there. Depending on the type of knowledge, this takes place in declarative (explicit) or non-declarative (implicit) memory. The retrieval of this information supports future learning and problem solving (Moreno and Mayer, 2007). Working memory plays a central role in the model. The CATLM assumes that working memory has a limited capacity, while long-term memory is considered unlimited. If the demands placed on learners exceed the available resources of the working memory, an overload occurs, which is referred to as “cognitive overload.” There is an overlap in content with the Cognitive Load Theory (CLT) by Sweller (1988), particularly in the area of evidence-based design of learning materials. Both models are based on the working memory model, which assumes limited attention resources when processing external representations. The learning benefit of materials is assessed based on the utilization of the working memory. The decisive factors here are the complexity of the learning material (intrinsic load), the didactic design (extraneous load) and the mental involvement of the learner (germane load).

However, the limitation of working memory is only subject to biologically secondary knowledge acquisition. CLT differentiates between biologically primary and secondary knowledge. Primary knowledge, such as speaking or facial recognition, is acquired intuitively and effortlessly, while secondary knowledge, such as reading or mathematics, requires explicit instruction and conscious effort, as it is not directly rooted in evolution (Sweller, 2020; Lespiau and Tricot, 2024; Kalyuga, 2023; Castro-Alonso et al., 2024). Relief can be achieved by externalizing internal processes to tactile (haptic) perceptions. For example, if a learner is supported by explicit instructions, visual representations, or interactive elements, fewer cognitive resources need to be used for the internal organization of information. This relieves the working memory and facilitates the acquisition of knowledge (Skulmowski and Xu, 2021). Similar assumptions are used in the EC approach and in theories and research on the embodiment principle. Embodied cognition (EC) states that sensory and motor systems influence cognitive processes (Hayes and Kraemer, 2017; Manches and O'Malley, 2012) and consequently, cognition cannot be separated from perception and action (Fiorella, 2021; Zou et al., 2025). If this is applied to the embodiment principle, it is assumed that students learn better when new concepts are explicitly linked to relevant actions, such as hand movements or the use of manipulatives. Embodiment while learning should reduce cognitive load and improve generative processing by linking new knowledge with analog actions (Glenberg, 2008; Novack and Goldin-Meadow, 2015). The working memory is thus relieved (Paas and Sweller, 2012). Studies show that haptic experiences of objects lead to detailed and lasting long-term memory representations, suggesting that touch is an important sensory channel for environmental information. Processing in working memory also integrates haptic experiences with information from other sensory channels (Hutmacher and Kuhbandner, 2018). In addition to CLT, it is considered that cognitive load is reduced through embodied instructions by enabling students to transfer their thoughts to their bodies, e.g., through gestures, or to the world by manipulating objects. This process effectively extends the capacity of working memory (Sepp et al., 2019). It should be mentioned that the distribution and focusing of attention is influenced by the learner's previous experiences as well as their current cognitive requirements, is interrelated, and is subject to constant change.

Summarizing these theoretical approaches, it can be assumed that rich sensory information also plays a central role in building a mental model of the learning object when learning with haptic models (manipulatives) and influences the learning process in terms of cognitive load, motivation, emotion and learning success.

The current state of empirical research shows that the inclusion of haptic models can significantly influence the overall learning success.

Several studies have explored the effects of learning with models across various subject areas, demonstrating that the use of physical and manipulable models can positively influence learning outcomes. The following table presents a selection of key research findings in this area.

As can be seen from Table 1, most empirical studies on learning with (3D) models primarily examine learning success, with models mainly used in STEM-related domains such as anatomy or engineering. Overall, the literature suggests that physical or 3D representations can support learning, particularly for content that involves complex spatial structures; however, effects are not necessarily uniform across topics, learner characteristics, and assessment formats. In addition, the outcome focus has been strongly performance-oriented: only one study assessed intrinsic motivation (Justo et al., 2022), one study examined cognitive load (Manrique et al., 2024), and one study assessed satisfaction (Chen et al., 2017). This indicates that emotional, cognitive, and motivational mechanisms that may explain when and why models are effective have rarely been examined systematically. Moreover, potential learner prerequisites and moderators have received limited attention. For example, only Stull et al. (2016) considered spatial ability, although such prerequisites may be crucial for learning from complex structures. Other relevant factors, such as individual interest, working memory capacity, or haptic learning preferences, have not yet been systematically investigated in this context. To address these gaps, the present study on two distinct anatomical domains (heart and spine) compares haptic models with closely matched visual materials and examines learning success as well as intrinsic motivation and cognitive load as key process outcomes, while additionally considering Need for Touch as a potential moderator.

**Table 1:** Summary of relevant studies with information on study field, target group, study design and measurement methods.

**Study**	**Field**	**Target group**	**Study design**	Measurements and key findings	
Stull et al. (2016)	Chemistry—molecular representations	University students	Study 1(*N =* 30): only models	Drawing accuracy	Main effect: not significant
				Spatial abilities (independent)^a^	Lower spatial abilities was associated with better drawing accuracy
			Study 2 (*N =* 64): models (only visual), models (manipulable), pictures	Drawing accuracy	Model manipulable > models (only visual) and pictures
				Spatial abilities (independent)^a^	No significant findings
			Study 3 (*N =* 59): models (only visual), models (manipulable), pictures	Drawing accuracy	Model manipulable > models (only visual) and pictures
				Spatial abilities (independent)^a^	No significant findings
Justo et al. (2022)	Engineering—structural kit	University students (*N =* 209)	Group 1: physical model Group 2: virtual model	Learning success	Not significant
			Intrinsic motivation^b^	Physical model > virtual model
			Attention^b^	Physical model > virtual model; for Relevance, Confidence
			Conceptual understanding	Only post: males > females
Krontiris-Litowitz (2003)	Neurophysiology	University students (*N =* 25)	Ion cell, nerve cell and membrane properties	Learning success	Model > no model
Reid et al. (2018), (qualitative study)	Anatomy—upper limb	Second—year medical students (*N =* 5)	Six-day educational intervention with a novel “haptico-visual observation and drawing” (HVOD) method	Self-reported learning success (retention)	Better memorization of knowledge
				Learning success (understanding)^c^	Improve cognitive understanding and memory
Kooloos et al. (2014)	Anatomy—brain	Second—year medical students	Study 1 (*N =* 128): Group 1: clay modeling Group 2: live observations	Learning success	Clay modeling < live observations
			Study 2 (*N =* 392): Group 1: clay modeling Group 2: video observations	Learning success	Clay modeling > video observations
Rau et al. (2015)	Medicine—Cleft lip and palate (CLP) model	University students (*N =* 138)	Practical tasks on the CLP model	Learning success	Improvement was achieved in 69.6%
Manrique et al. (2024)	Anatomy—scapulae, humerus and clavicle	Second—year medical students (*N =* 85)	Group 1: 2D method Group 2: 3D plaster bone model Group 3: no workshop	Learning success	2D method < 3D model
				Cognitive load (self—constructed)	Understanding anatomical landmarks: 2D method < 3D model
				Experience with model	2D method < 3D model
Tanner et al. (2020)	Anatomy-−3D printed model of the pterygopalatine fossa (PPF)	Students (*N =* 118)	Study 1: prior knowledge of PPF Group 1: half skull Group 2: 3D model Study 2: no prior knowledge Group 1: half skull Group 2: 3D model	Learning success	Group 1: half skull < 3D model Group 2: half skull < 3D model (not significant but improvement)
				Satisfaction (self—constructed)	Group 1 and 2: half skull < 3D model
Chen et al. (2017)	Anatomy–colored 3D skull model	Medical students (*N =* 79)	Group 1: 3D skull model Group 2: cadaveric skulls Group 3: atlas (anatomic)	Learning success	3D skull model > cadaveric skulls and atlas (post-test)
Cai et al. (2019)	Anatomy–3D printed knee model	Medical students (*N =* 35)	Group 1: 3D model for each student Group 2: 3D model in front of class (“didactic learning”)	Learning success	3D model for each student > 3D model in front of class
Kong et al. (2016)	Anatomy–3D printed model of hepatic structures	Medical students (*N =* 92)	Group1: 3D models Group 2: atlas (anatomic) three different types of models	Learning success	3D models (each type) > atlas

^a^Vandenberg and Kuse (1978).

^b^Keller (2009).

^c^13 items test based on the specific learning objectives (Reid et al., 2018).

## Research questions and hypotheses

The study aimed to investigate the influence of haptic learning materials on learning outcomes, intrinsic motivation, and cognitive load in two different domains and learning materials (heart and spine). Specifically, the study sought to understand how individual differences in the Need for Touch (NFT) might affect these variables when learning with haptic vs. non-haptic (visual) materials.

The research was structured around the following hypotheses:

- **Higher Learning Success (H1):** Learners using haptic materials would achieve greater learning success compared to those using pictorial materials.
- **Higher Intrinsic Motivation (H2):** Learners using haptic materials would report higher intrinsic motivation.
- **Lower Cognitive Load (H3):** Learners using haptic materials would experience a lower cognitive load.

## Method

### Research design

This study employed an experimental design to investigate the effects of haptic learning materials on learning success, intrinsic motivation, and cognitive load. The study procedure began with the preparation phase, during which participants received an introduction to the study protocol and signed an informed consent form. In the pre-test phase, baseline data were collected through questionnaires that covered sociodemographic information, Need for Touch (NFT) for learning, individual interest (Luo et al., 2019), and prior knowledge about the heart and spine.

NFT was assessed as an individual-difference variable (not to form groups) to capture potential preferences for tactile information processing that may moderate the effects of haptic vs. visual materials, given the limited empirical evidence in educational haptic-learning contexts.

The study used a counterbalanced within-subject crossover design: each participant completed one visual and one haptic learning phase and learned both condition (heart and spine). Topic order and modality order were counterbalanced across participants via four sequence conditions. Participants completed two learning phases (15 min each). Participants were approximately evenly distributed across the four order conditions.

**Figure 2:**
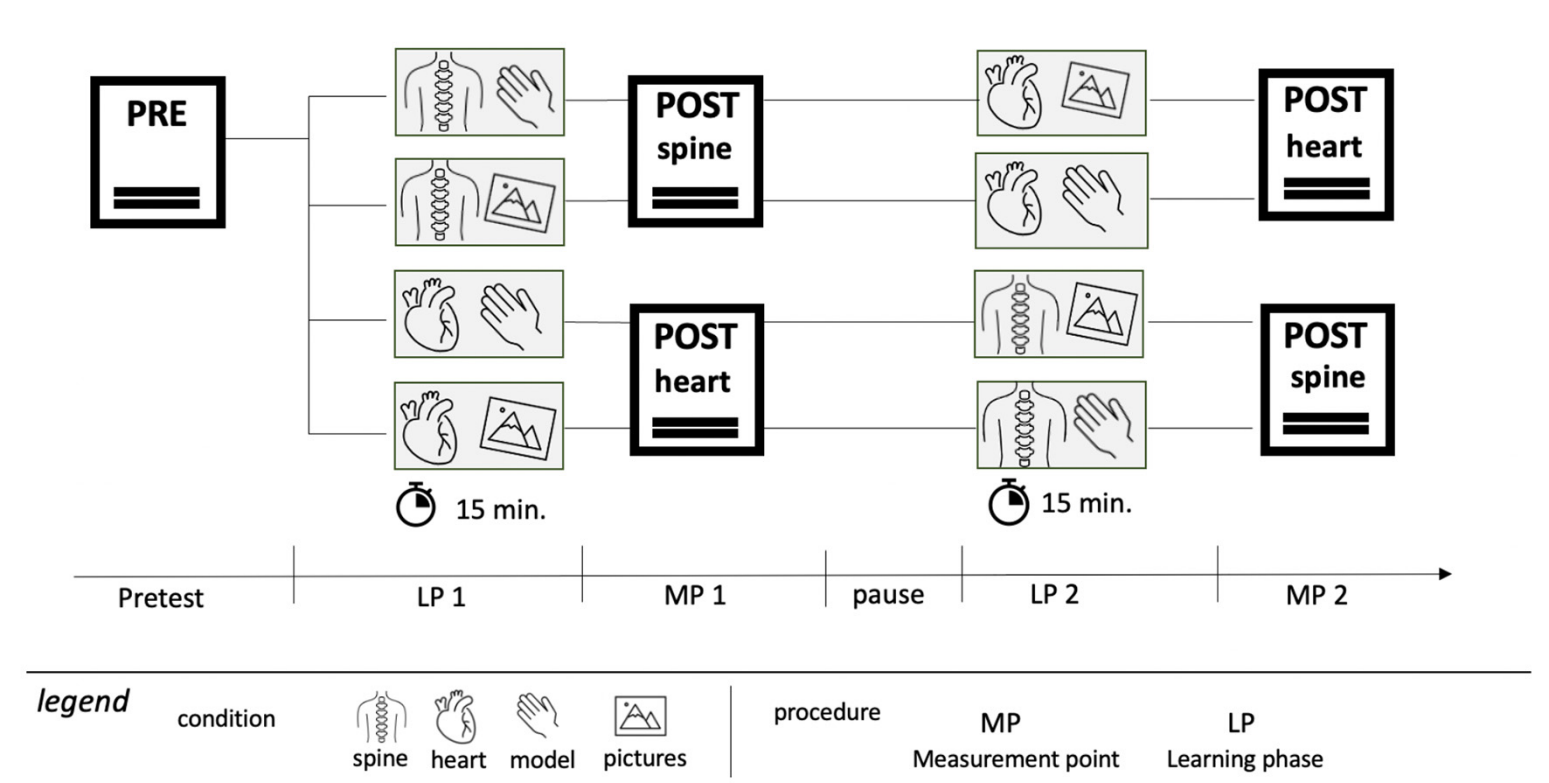
Study design.

As can be seen in Figure 2, the study participants were either first given the learning material with images of the spine and then, in the second round, the learning material with the model of the heart, or the other way round. During each learning phase, participants additionally received unpictured informational texts on the respective topic to standardize the informational content across conditions. Following the learning session, the post-test phase involved assessing cognitive load (Klepsch et al., 2017), intrinsic motivation (Isen and Reeve, 2005) and learning outcomes through a knowledge test on the heart and spine. Additionally, qualitative data on the learning process were collected to provide further insights into the participants' experiences. The mixed running order between learning materials and presentation formats was used to control for effects of the order of task format.

### Material and measures

#### Learning material

The learning content covered two anatomical topics (heart and spine). Heart and spine anatomy were selected because they are relevant to students' training, are generally familiar at a basic level without requiring extensive prior knowledge and typically elicit interest. In each learning phase, all participants received the same written learning text (without pictures).

Depending on condition, the text was supplemented either by visual materials (pictures) or by a physical anatomical model that could be handled and explored by touch. In the visual condition, participants received four photographs per topic showing the corresponding model from different perspectives.

In the haptic condition, participants handled the physical models directly. The heart model could be opened, whereas the spine topic was represented by two separate models (a spine model and an individual vertebra model).

**Figure 3:**
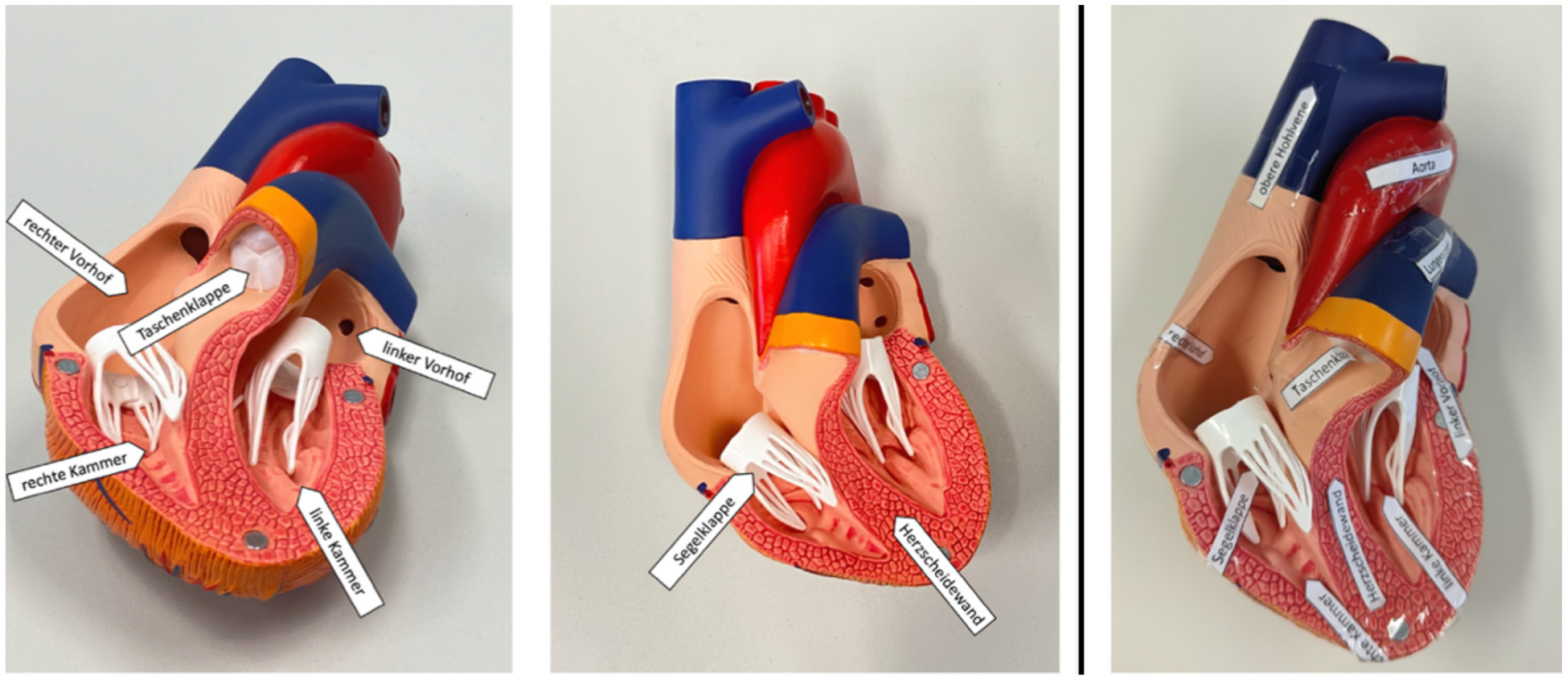
Heart learning materials. **Left:** examples of the visual materials (two photos of the model). **Right:** the physical model used in the haptic condition (labeled structures).

**Figure 4:**
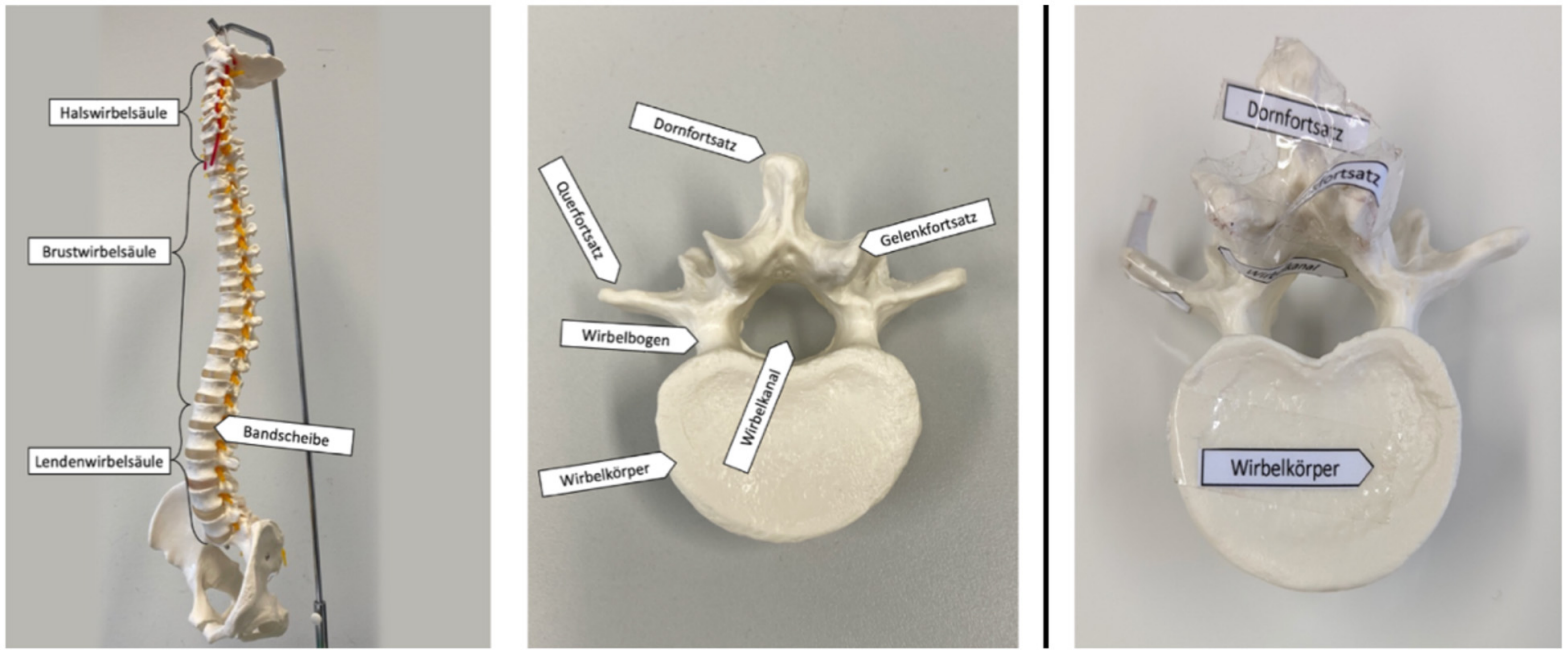
Spine learning material. **Left:** examples of the visual materials (two photos of the model). **Right:** one of the physical model used in the haptic condition (labeled structures).

Both models were labeled with the corresponding anatomical structures. The anatomical models were purchased from a teaching model manufacturer (“Erler-Zimmer”) and the visual materials consisted of photographs depicting the corresponding models from different perspectives to ensure that all information was comparable across the visual and haptic conditions. Figures 3, 4 show the picture and model materials for the heart and spine topics, respectively.

#### Need for Touch

The “Need for Touch Scale” (NFT) (German version: Nuszbaum et al., 2012) is a psychometric tool developed by Peck and Childers (2003), which assesses individual differences in the preference and necessity for tactile interaction with objects.

This scale originally emerged in the field of consumer research to explore how haptic (touch-related) information influences purchasing decisions and is divided into two subscales: Instrumental Need for Touch (1), this aspect relates to the functional purpose of touch, where individuals seek tactile input to obtain information about a product to aid in decision-making. Autotelic Need for Touch (2), this dimension captures the enjoyment and satisfaction derived from the act of touching itself, independent of any practical outcome.

Recent research has extended the application of the NFT Scale to educational contexts, particularly to understand how tactile interactions with learning materials might influence learning outcomes. The scale was adapted to create the “Need for Touch—Learning Scale,” which includes items reflecting how touch can aid in understanding learning content. The adaptation involved transforming statements related to product interaction into educational scenarios. For instance, the statement “I feel more comfortable purchasing a product after physically examining it” was adapted to “I feel more comfortable in being able to understand the learning content correctly if I can touch it while learning.” A pilot study with 20 participants confirmed the comprehensibility of the adapted scale.

Based on test-theoretical analyses conducted in the main study (*N* = 117), nine items were retained for the final version of the scale, and the original “Demand” and “Preference” subscales were not used further. In the validation study by Büscher et al. (2023), the total scale showed excellent internal consistency with a Cronbach's alpha value of 0.953. In our sample, the adapted scale also demonstrated high internal consistency of 0.872. All items are provided in the Supplementary material S1.

#### Individual interest

The Academic Interest Scale for Adolescents (AISA; Luo et al., 2019) measures students' individual academic interest across different domains and is based on the four-phase model of interest development by Hidi and Renninger (2006). It comprises four subscales: emotion, value, knowledge, and engagement. In the original validation study, internal consistency was high, with Cronbach's alpha values of 0.87, 0.90, 0.88, and 0.86 in mathematics, and 0.90, 0.93, 0.93, and 0.91 in English, respectively. In our study, the instrument was adapted in terms of content and language to fit the context of anatomical learning materials. The internal consistency of the subscales in our sample (*N* = 87) was also satisfactory: emotion (0.930), value (0.834), knowledge (0.878), and engagement (0.896).

#### Cognitive load

The Cognitive Load Questionnaire developed by Klepsch et al. (2017) captures all three types of cognitive load as defined in cognitive load theory: intrinsic cognitive load (ICL), extraneous cognitive load (ECL), and germane cognitive load (GCL). The instrument consists of eight items rated on a 7-point Likert scale, distributed across three subscales. ICL refers to the complexity inherent in the learning material, ECL to cognitive strain caused by instructional design, and GCL to the effort invested in understanding and schema construction. Example items include “This task was very complex” (ICL), “The design of this task was very inconvenient for learning” (ECL), and “My point while dealing with the task was to understand everything correctly” (GCL). In the original validation study, internal consistencies with Cronbach's alpha values were 0.86 (ICL), 0.80 (ECL), and 0.80 (GCL).

In our study, reliability analyses were conducted separately for each learning material (heart vs. spine) and representation format (haptic vs. visual), due to the within-subjects design. Internal consistency values varied across conditions. For the heart material, Cronbach's alpha values were 0.810 (ICL), 0.755 (ECL), and 0.553 (GCL); for the spine material, values were 0.571 (ICL), 0.637 (ECL), and 0.647 (GCL).

#### Intrinsic motivation

The Intrinsic Motivation Scale by Isen and Reeve (2005) assesses the extent to which individuals engage in an activity for its own sake, due to inherent enjoyment and interest, rather than external incentives such as rewards or social pressure. The scale comprises eight items, such as “It is enjoyable” and “It makes me feel curious about it,” rated on a 7-point Likert scale. In the original validation studies, the scale demonstrated excellent internal consistency, with Cronbach's alpha values of 0.92 (interesting puzzle task) and 0.93 (uninteresting letter string task).

In our study, the scale was applied separately for two types of learning materials (heart vs. spine) and two presentation formats (haptic vs. visual). Internal consistency was excellent in the heart condition (0.968) as well as in the spine condition (0.963).

#### Learning tests

The prior knowledge and learning tests consisted of both closed—and open-ended items, with varying scoring schemes, in order to gather information about visual, verbal, spatial and haptic knowledge. While learning test items had a fixed maximum score, some prior knowledge items involved open responses with no predefined maximum (e.g., “Name all components of the heart you can think of,” with one point per correct element). To ensure consistency across instruments, internal consistency was assessed using Cronbach's alpha for all four tests.

#### Prior knowledge

To assess participants' prior knowledge, two domain-specific tests were self-developed, one on the spine (6 questions) and one on the heart (5 questions). The tests differed slightly in length to ensure adequate coverage of the key concepts for each topic.

The tests included both closed and open-ended items. The full item set is provided in Supplementary material S2 and S3. Internal consistency with Cronbach's alpha values was measured with 0.472 for the heart condition and 0.356 for the spine condition.

#### Learning test

Two learning tests were developed to assess participants' learning progress, one focused on the spine and one on the heart. The questions targeted different levels of cognitive processing, including retention and comprehension, and addressed both textual and visual information. The spine test comprised 13 questions (8 retention, 5 comprehension; max. 35 points). An example item for retention was: “How many movable and how many rigid vertebrae does the spine have?”). while a comprehension item was “Why do adults get smaller as they get older?”.

The heart test included 9 questions (5 retention, 4 comprehension; max. 21 points). Sample items were: “Through which artery is the blood distributed from the heart throughout the body?” (retention) and “How can it happen that the heart is not sufficiently supplied with energy?” (comprehension). The full item set is provided in Supplementary material S4 and S5.

Items were scored using predefined scoring rules; open-ended responses received points based on the number of correct elements mentioned.

Internal consistency with Cronbach's alpha values was measured with 0.435 for the heart condition and 0.653 for the spine condition. The heart and spine learning tests differed slightly in length and maximum score because they were developed to match the topic-specific content breadth and complexity and to ensure adequate coverage of the learning objectives for each topic. Comprehension items were designed to require integration of information from the learning text and the respective topic materials; they were not intended to be attributable to a single modality. Importantly, the heart and spine learning tests were not intended to be compared directly across topics. Instead, our aim was to identify motivational and cognitive load related patterns of effects of different haptic learning materials. Therefore, our primary analyses compare visual vs. haptic conditions within each topic using the same topic-specific test, and results are therefore reported separately for heart and spine.

#### IRT-based analysis of the test instruments

To check the psychometric quality of the knowledge and learning tests used, analyses based on item response theory were carried out for all four instruments (prior knowledge heart, prior knowledge spine, learning test heart, learning test spine) using the snowIRT module in jamovi. A partial credit model (PCM) was used for each of the prior knowledge tests, as the items had graded response categories (e.g., differentiated descriptive elements). The learning tests were analyzed using the dichotomous Rasch model due to their dichotomous response distributions. IRT analyses were used to evaluate item functioning and test reliability.

The person separation reliability was 0.39 in the heart prior knowledge test, 0.39 in the spine prior knowledge test, 0.75 in the heart learning test and 0.71 in the spine learning test. The comparatively low reliability of the prior knowledge tests is presumably due to the limited number of items and the heterogeneous, open response format, combined by heterogenous levels of prior knowledge. However, these instruments were primarily designed to enable a rough classification of the learners' prior conceptual knowledge. The learning tests, on the other hand, showed satisfactory reliability and are therefore suitable for differentiated assessment of performance levels after instruction.

The item fit statistics were predominantly within the accepted range (infit/outfit between 0.7 and 1.3); individual items with slight deviations were retained for reasons of content in order to ensure the structural validity of the tests. In all four instruments, the Wright maps showed an appropriate fit between the item difficulties and the distribution of personal abilities.

### Participants

In the study conducted at a German university of applied science, the participants primarily comprised university students specializing in psychology from the same degree programme, semester and course. The total sample size was 87 students, with a significant gender disparity (78 females and 9 males). Participants ranged in age from 18 to 29 years, with an average age of 20.72 years (SD = 2.276).

## Results

We first tested for potential order effects (topic order and modality order). After checks confirmed that no ordering effects occurred, we combined the data for each condition, irrespective of the order of presentation. Data were pooled accordingly to topic and modality for later data analyses (SPINE_vis_ and SPINE_hapt_ as well as HEART_vis_ and HEART_hapt_).

Next, we examined whether there are systematic differences with regard to the learning requirements prior knowledge, individual interest and NFT (Tables 2–5). The results are shown in the following tables. In addition to *t-*values and *p-*values, effect sizes as Cohen's d were reported together with 95% confidence intervals. This provides information on the magnitude and precision of effects beyond statistical significance.

**Table 2:** Means, standard deviation (SD), *t-*values, *p-*values, effect sizes (Cohen's d), and 95% confidence intervals (CI) for prior knowledge.

**Learning content**	**Visual**	**Haptic**	***t*-test**
	Mean (SD)	Mean (SD)	
HEART	45.36 (18.16)	40.93 (20.19)	*t*_(85)_ = −1.07, *p =* 0.289, 95% CI (−0.66, 0.19), d = −0.24
SPINE	32.64 (11.38)	34.41 (11.96)	*t*_(85)_ = 0.71, *p =* 0.481, 95% CI (−0.28, 0.57), d = 0.15

**Table 3:** Means, standard deviation (SD), *t-*values, *p-*values, effect sizes (Cohen's d), and 95% confidence intervals (CI) for NFT and individual interest between the two conditions.

**Measure**	**HEART_vis/_SPINE_hapt_**	**SPINE_vis/_HEART_hapt_**	***t*-test**
	Mean (SD)	Mean (SD)	
Need for Touch	2.82 (0.70)	2.69 (0.73)	*t*_(85)_ = 0.81, *p =* 0.421, 95% CI (−0.60, 0.25), d = −0.17
Individual interest	3.09 (0.71)	3.08 (0.63)	*t*_(85)_ = 0.08, *p =* 0.939, 95% CI (−0.44, 0.41), d = −0.02

**Table 4:** Means, standard deviation (SD), *t-*values, *p-*values, effect sizes (Cohen's d), and 95% confidence intervals (CI) for learning success, intrinsic motivation, and cognitive between visual and haptic for the condition heart.

**HEART**	**Visual**	**Haptic**	***t-*test**
	Mean (SD)	Mean (SD)	
**Learning success**			
Total	11.57 (4.21)	12.38 (3.84)	*t*_(85)_ = 0.94, *p =* 0.176, 95% CI (−0.22, 0.62), d = 0.20
Comprehension	1.97 (1.16)	1.80 (1.07)	*t*_(85)_ = −0.69, *p =* 0.245, 95% CI (−0.57, 0.27), d = 0.15
Picture retention	7.12 (3.15)	8.19 (2.87)	*t*_(85)_ = 1.65, *p =* 0.051, 95% CI (−0.07, 0.78), d = 0.36
Text retention	2.47 (0.905)	2.38 (1.13)	*t*_(85)_ = −0.41, *p =* 0.340, 95% CI (−0.51, 0.33), d = −0.09
**Intrinsic motivation**			
Intrinsic motivation	4.51 (1.45)	4.56 (1.59)	*t*_(85)_ = 0.15, *p =* 0.880, 95% CI (−0.39, 0.45), d = 0.03
**Cognitive load**			
Intrinsic	4.83 (1.30)	4.76 (1.47)	*t*_(85)_ = 0.23, *p =* 0.817, 95% CI (−0.47, 0.37), d = −0.05
Extraneous	3.44 (1.39)	2.52 (1.05)	*t*_(85)_ = 3.54, *p* < 0.001, 95% CI (−1.12, −0.32), d = −0.76
Germane	6.02 (0.74)	6.40 (0.61)	*t*_(85)_ = 2.70, *p =* 0.008, 95% CI (0.15, 1.01), d = 0.58

**Table 5:** Means, standard deviation (SD), *t-*values, *p-*values, effect sizes (Cohen's d), and 95% confidence intervals (CI) for learning success, intrinsic motivation, and cognitive between visual and haptic for the condition spine.

**SPINE**	**Visual**	**Haptic**	***t-*test**
	Mean (SD)	Mean (SD)	
**Learning success**			
Total	23.39 (4.13)	23.30 (4.50)	*t*_(85)_ = −0.09, *p =* 0.465, 95% CI (−0.47, 0.41), d = −0.02
Comprehension	5.41 (1.37)	5.58 (1.46)	*t*_(85)_ = 0.57, *p =* 0.284, 95% CI (−0.30, 0.55), d = 0.13
Picture retention	11.44 (2.58)	11.50 (2.73)	*t*_(85)_ = 0.09, *p =* 0.463, 95% CI (−0.40, 0.44), d = 0.02
Text retention	6.59 (1.49)	6.30 (1.38)	*t*_(85)_ = −0.95, *p =* 0.172, 95% CI (−0.63, 0.22), d = −0.21
**Intrinsic motivation**			
Intrinsic motivation	4.40 (1.42)	5.40 (1.38)	*t*_(85)_ = 3.30, *p =* 0.001, 95% CI (0.27, 1.14), d = 0.71
**Cognitive load**			
Intrinsic	3.82 (1.32)	3.55 (1.29)	*t*_(85)_ = 0.96, *p =* 0.341, 95% CI (−0.63, 0.22), d = −0.21
Extraneous	2.37 (0.98)	2.15 (1.08)	*t*_(85)_ = 0.99, *p =* 0.325, 95% CI (−0.64, 0.21), d = −0.21
Germane	6.08 (0.77)	6.27 (0.74)	*t*_(85)_ = 1.16, *p =* 0.250, 95% CI (−0.18, 0.67), d = 0.25

### Prior knowledge, individual interest and Need for Touch

As the NFT was only measured once per test subject and each test subject completed both learning units, the following table differentiates between the two conditions (irrespective of order: HEART_vis/_SPINE_hapt_ vs. SPINE_vis/_HEART_hapt_) according to which learning material was presented as a model or illustration.

### Learning success, intrinsic motivation and cognitive load

To investigate the main effects, *t-*tests were used. To examine the influence of the Need for Touch, linear regressions were conducted for each of the two groups using the NFT as a predictor. The results for both learning materials and presentation formats are presented in the following tables.

Statistical analysis revealed no effects of the presentation format (visual vs. haptic) regarding learning success and intrinsic motivation. For different types of cognitive load, we found the following pattern: Test persons reported higher extraneous load in the visual than in the haptic condition, whereas germane load resulted higher in the haptic than in the visual condition. That shows that the irrelevant extraneous load is reduced through the haptic material while the important germane load conducive to comprehension is increased.

In contrast to the heart material statistical analysis showed no effects of the presentation format (visual vs. haptic) regarding learning success and cognitive load. Moreover, contrastingly an effect of the presentation format on intrinsic motivation with higher values in the haptic condition was revealed. The results are mixed and not completely consistent for both learning contents but taken together results show the potential of haptic learning material for promoting learning via favorable pattern of cognitive load and raised intrinsic motivation.

### Influence of the Need for Touch

To analyze the relation of the Need for Touch and cognitive load (heart) as well as intrinsic motivation (spine) a regression analysis was conducted. Both analysis showed a significant model fit. Results are visualized in the following graphics.

**Figure 5:**
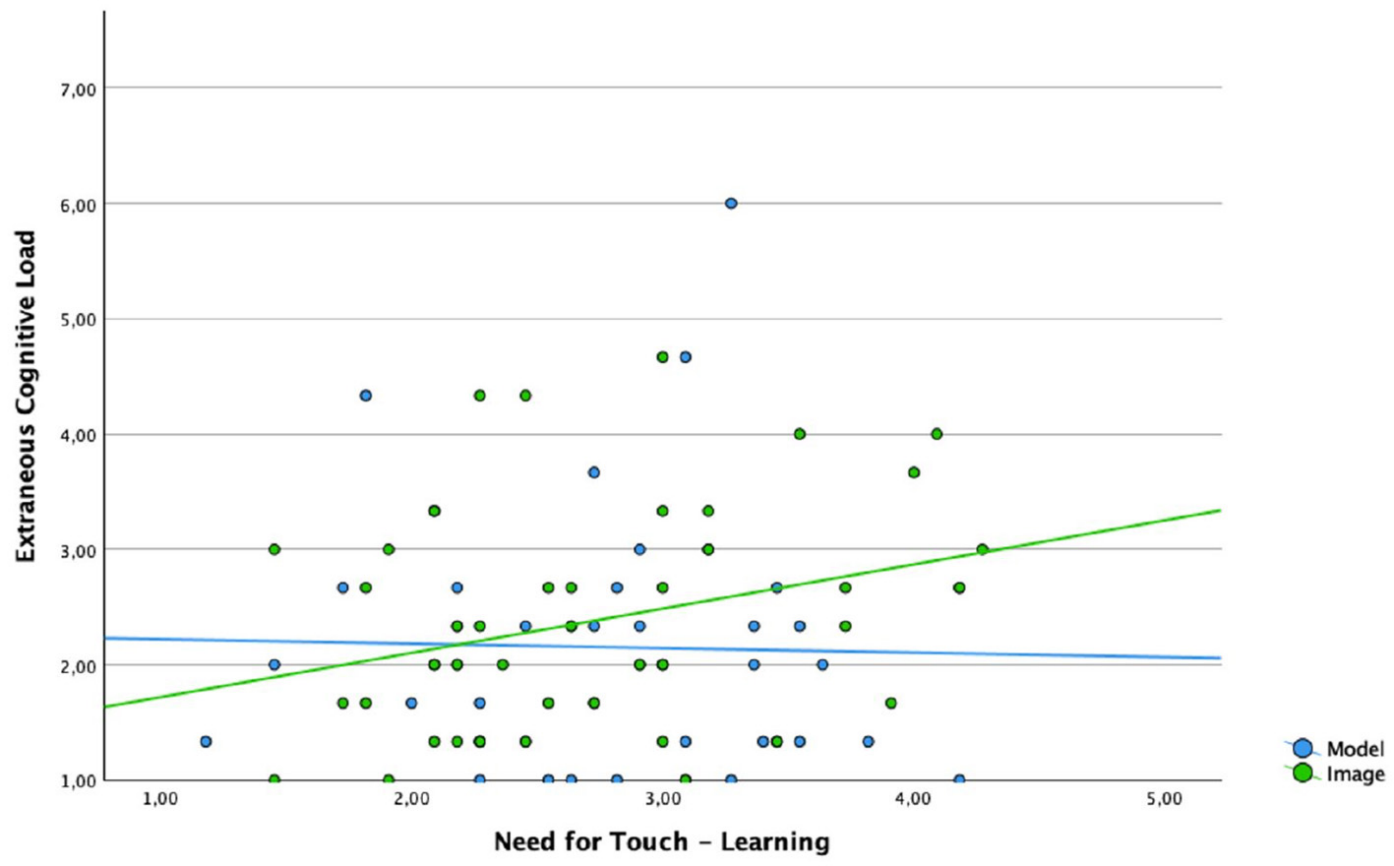
Linear regression between extraneous cognitive load and NFT for the condition visual and haptic (heart material).

As displayed in Figure 5 Need for Touch predicts the extraneous load reported in the visual heart material (R^2^ = 0.083, F_(1, 45)_ = 4.07, *p* = 0.050): The higher the Need for touch, the more the extraneous load increases when learners are confronted with visual heart material. In contrast when learners use the haptic version of the material there is no relation between need for touch and extraneous load (R^2^ = 0.001, F_(1, 8)_ = 0.02, *p* = 0.878).

**Figure 6:**
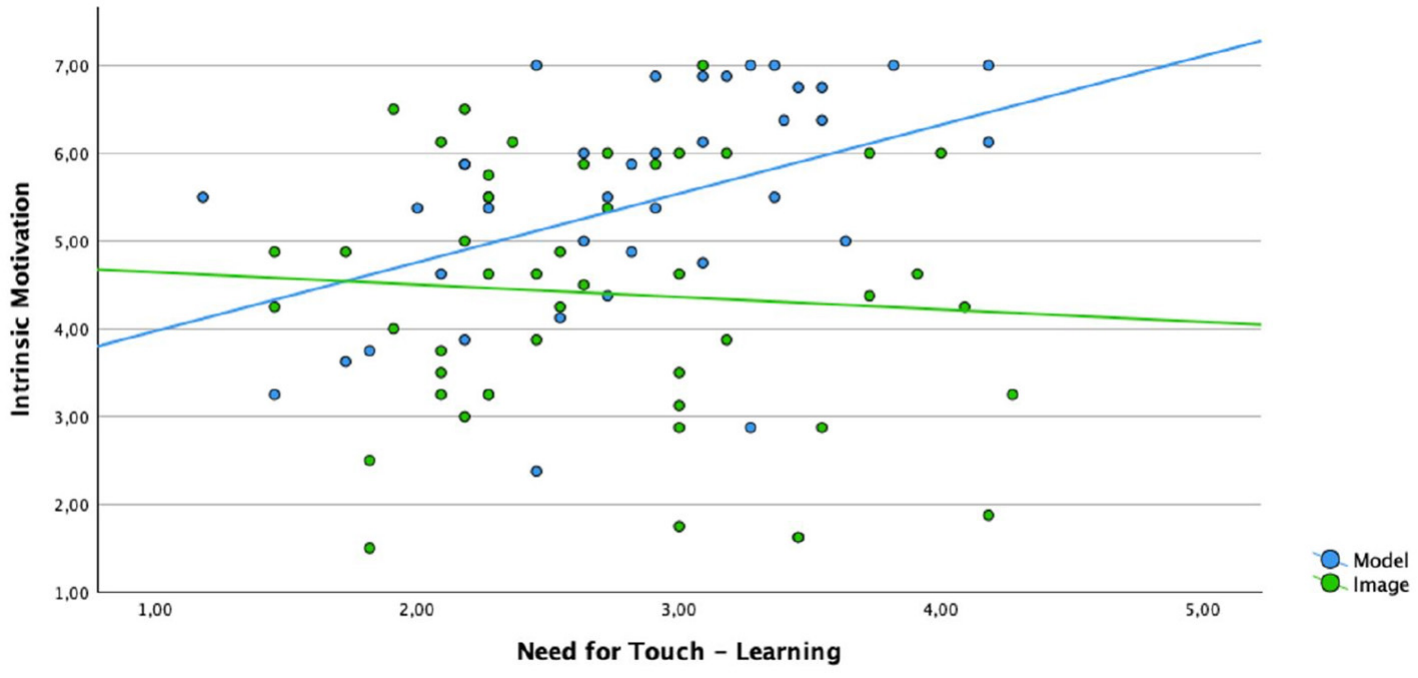
Linear regression between intrinsic motivation and NFT for the condition visual and haptic (spine material).

As can be seen in Figure 6: For the spine material the following relation was found. The higher the Need for Touch, the more intrinsic motivation rises when learners are presented with the haptic material (R^2^ = 0.158, F_(1, 38)_ = 7.13, *p* = 0.011). In opposite when learners view the visual material this relation does not occur (R^2^ = 0.005, F_(1, 45)_ = 0.29, *p* = 0.627).

### Germane load

No systematic correlations between NFT and germane load could be found for the learning material on the heart (haptic: R^2^ = 0.003, F_(1, 45)_ = 0.14, *p* = 0.711; visual: R^2^ < 0.001, F_(1, 38)_ = 0.02, *p* = 0.898).

## Summary and discussion

This study investigated the effects of haptic compared to visual learning materials on learning success, intrinsic motivation, and cognitive load, and further examined individual differences in Need for Touch (NFT). Overall, no significant differences between presentation formats were found for learning success (H1). A reason for this null effect on learning success might be that modality effects may not always translate into immediate performance gains, particularly in short learning phases or when assessments capture broad, topic-specific knowledge. From a CLT/CTML perspective, haptic support may primarily influence learning indirectly via processing efficiency and engagement rather than producing uniform performance advantages across contents (Sweller, 2020; Mayer, 2005; Moreno and Mayer, 2007). However, the pattern of effects differed depending on the learning content: for the spine topic, intrinsic motivation differed between conditions (H2), whereas for the heart topic, differences emerged primarily in cognitive load (H3). Regarding motivation, the content-specific motivational effect aligns with CATLM's emphasis on affective pathways in learning and suggests that embodied interaction may enhance situational engagement depending on topic characteristics and perceived relevance (Moreno and Mayer, 2007; Fiorella, 2021).

In terms of cognitive load, participants reported higher extraneous load in the visual heart condition compared to the haptic condition, suggesting that handling a physical model may reduce irrelevant processing demands. According to CLT, this pattern is compatible with the interpretation that haptic exploration can reduce extraneous processing by providing information in a more directly perceivable format, thereby freeing resources for learning-relevant processing (Paas and Sweller, 2012; Sweller, 2020). In addition, germane load tended to be higher (on a descriptive level) in the haptic heart condition, indicating that haptic materials while reducing extraneous load at the same time may foster learning-relevant processing.

We focused on cognitive load and intrinsic motivation because prior work on haptic and embodied learning often prioritizes performance outcomes, while the underlying cognitive and affective mechanisms are less frequently assessed. In line with CATLM and cognitive load theory, motivation and cognitive load were therefore selected as key process variables that may explain why and under which conditions haptic materials support learning, even when immediate performance differences are not observed.

### Differences between learning contents (heart vs. spine)

Across outcomes, the pattern of effects differed between the two learning contents. Specifically, for the heart condition, the comparison of visual vs. haptic learning materials yielded a significant effect on cognitive load, most notably on extraneous cognitive load. In contrast, for the spine condition, the most pronounced difference between conditions emerged for intrinsic motivation. Importantly, because the heart and spine learning tests were topic-specific and differed in length and maximum score, we primarily interpret effects within each topic (visual vs. haptic) rather than as direct cross-topic comparisons of raw test scores. One plausible explanation relates to differences in content and model characteristics and cognitive demands. The spine topic is strongly structure—and relation-based (segmental organization, spatial relationships), whereas the heart topic additionally involves more functional and process-related aspects. Moreover, the spine model might be more impressive and unusual as it is above one meter high on a typical table (skeleton), while the heart model can be opened and can be examined trough more complex exploration procedures. Such differences may influence which modality provides the most useful support and whether benefits emerge primarily in motivational engagement or in reduced processing demands (e.g., lower extraneous load). This interpretation is consistent with prior work suggesting that the effectiveness of embodied or object-based supports depends on task demands and how well the representation affords the required spatial or functional inferences (Skulmowski and Xu, 2021; Castro-Alonso et al., 2024).

A second explanation concerns the affordances of the learning materials. The heart model could be opened, potentially facilitating access to internal structures and supporting integration with less extraneous processing. In contrast, the spine topic was represented by two separate models (spine and an individual vertebra), which may have shaped how learners explored and integrated information across representations. Together, these topic—and material-specific characteristics may help explain why the heart and spine results diverged, even though the overall instructional approach was comparable. Moreover, the comparatively low internal consistencies observed for some knowledge tests should be interpreted cautiously, as such tests often cover heterogeneous content facets and may function as formative measures rather than reflecting a single homogeneous construct, which can naturally result in lower Cronbach's alpha values (Zitzmann and Orona, 2025; Stadler et al., 2020).

Regarding individual differences, regression analyses indicated that NFT was related to extraneous load in the visual heart condition: higher NFT was associated with higher extraneous load when learning from pictures, whereas this association was not observed in the haptic condition. In addition, for the spine topic, higher NFT was associated with higher intrinsic motivation in the haptic condition only. Taken together, these findings suggest that learners with a high NFT may particularly benefit from haptic materials, as physical models may reduce extraneous cognitive load and increase motivational engagement depending on the learning content and outcome considered. This moderation pattern is in line with the conceptualization of NFT as a preference for tactile information acquisition, implying that haptic materials may be particularly beneficial when they match learners' sensory preferences (Peck and Childers, 2003; Büscher et al., 2023).

### Theoretical implications

The findings contribute to the theoretical understanding of learning with multiple representations by extending established models such as Cognitive Load Theory (CLT; Sweller, 1988, 2020), the Cognitive Theory of Multimedia Learning (CTML; Mayer, 2005), and the Cognitive-Affective Theory of Learning with Media (CATLM; Moreno and Mayer, 2007). They suggest that haptic interaction may represent an additional modality that complements these frameworks by adding an embodied, sensorimotor dimension to multimodal learning (Fiorella, 2021; Zou et al., 2025).

Rather than indicating uniform performance gains, our results point to content—and outcome-specific influences of haptic learning: in our data, haptic materials were primarily associated with lower extraneous cognitive load for the heart topic and higher intrinsic motivation for the spine topic. This pattern is consistent with the view that tactile experiences may support learning indirectly by shaping processing efficiency and motivational engagement, depending on the content and task characteristics (Castro-Alonso et al., 2024; Kalyuga, 2023; Lespiau and Tricot, 2024). In line with embodied cognition accounts, sensory experiences may externalize aspects of cognitive processing and thereby reduce working-memory demands under certain conditions (Skulmowski and Xu, 2021; Fehrmann, 2025).

### Methodological implications

While the sample size of this study is solid, future research would benefit from larger and more heterogeneous samples (e.g., broader variation in study background, gender, age, and educational attainment) to increase statistical power and generalizability. In addition, further work should examine relevant learner characteristics and cognitive prerequisites (e.g., spatial ability) that may shape how learners benefit from haptic models (Stull et al., 2016; Manrique et al., 2024).

A deeper understanding of haptic learning processes may require complementing quantitative outcomes with process-oriented methods. Mixed-method approaches could clarify how learners explore and integrate haptic information (e.g., handling strategies and perceived object properties such as weight or material features) and how these processes relate to cognitive load, motivation, and learning success (Ratcliffe and Tokarchuk, 2020; Fehrmann, 2025; Zou et al., 2025). Moreover, because NFT is still a relatively novel construct in this context, future studies should further evaluate its reliability and applicability across diverse target groups beyond predominantly female student samples (Büscher et al., 2023).

### Practical implications

The present findings suggest that incorporating haptic models may support learning processes in domains that involve complex spatial and structural content (e.g., STEM and health-related education). In our study, haptic materials were associated with lower extraneous cognitive load for the heart topic and higher intrinsic motivation for the spine topic, indicating that physical interaction with models can facilitate processing efficiency and engagement under certain conditions (Paas and Sweller, 2012; Sweller, 2020; Fehrmann, 2025). Rather than implying uniform performance gains, these results highlight that the benefits of haptic materials may be content—and outcome-specific.

To make haptic learning more accessible, low-cost physical models, including, where feasible, 3D-printed models, may represent a practical option for educational settings (Chen et al., 2017; Tanner et al., 2020). However, the effectiveness of such implementations likely depends on the instructional context and on how well the models are aligned with the learning objectives and accompanying materials (Mayer and Moreno, 2003; Moreno and Mayer, 2007). In addition, individual differences such as Need for Touch (NFT) may be relevant when selecting or designing learning materials, as learners with a high NFT may particularly benefit from opportunities for hands-on exploration (Peck and Childers, 2003; Büscher et al., 2023).

### Limitations

A limitation concerns the comparatively low internal consistency observed for some measures (e.g., α ≈ 0.42). This may reflect the content diversity and more formative character of the tasks rather than a lack of psychometric quality; nevertheless, it should be considered when interpreting the results. In addition, the heart and spine tests were topic-specific and not fully parallel in length and scoring, which limits direct cross-topic comparisons. Future studies could address this by using more tightly parallelized item sets and psychometric equating (e.g., parallel forms or IRT-based linking) to improve measurement precision and comparability across topics (Sweller, 2020).

Finally, one outcome approached conventional significance (Retention Picture task; *p* = 0.051). This finding should be interpreted cautiously and warrants replication with larger samples and/or more sensitive analytical approaches.

### Future research

Future work should further clarify under which conditions haptic materials are most beneficial by systematically varying learning content and task demands. Given the content-specific pattern observed here (heart: extraneous cognitive load; spine: intrinsic motivation), future studies should use more tightly parallelized assessment instruments and transparent scoring rules and, where appropriate, psychometric equating to facilitate comparisons across topics and outcomes (Sweller, 2020; Skulmowski and Xu, 2021). Future studies could extend this approach by examining additional evaluative and contextual variables, such as perceived usefulness, social/contextual factors, or other potential mediators and moderators, to better understand when and for whom haptic materials are most beneficial.

In addition, process-oriented approaches are needed to better understand how learners explore and integrate haptic information. Mixed-method and process measures (e.g., observation/video-based coding, think-aloud protocols, or eye-tracking) could help identify exploration strategies and attention allocation patterns that may explain differences in cognitive load and motivation (Mayer and Moreno, 2003; Moreno and Mayer, 2007). Individual differences such as Need for Touch (NFT) should be examined in larger and more diverse samples to test whether the observed moderation effects generalize (Peck and Childers, 2003; Büscher et al., 2023).

Finally, an important next step is to test haptic learning in more ecologically valid settings, such as longer instructional units and delayed post-tests. Building on frameworks that integrate cognitive and affective processes in immersive environments (e.g., CATLM and related extensions), future research may also explore whether and how haptic interaction in digital or virtual learning settings contributes to cognitive load, motivation, and learning outcomes (Moreno and Mayer, 2007; Makransky and Lilleholt, 2018).

## Data Availability

The raw data supporting the conclusions of this article will be made available by the authors, without undue reservation.

## References

[B1] Ainsworth S. (2006). DeFT: a conceptual framework for considering learning with multiple representations. Learn. Instr. 16, 183–198. doi: 10.1016/j.learninstruc.2006.03.001

[B2] Büscher L. Montag M. Zander S. (2023). “*Need for touch in learning (NFT-L): entwicklung eines fragebogens zu haptischen lernpräferenzen*,” in PAEPS-Tagung (Jahrestagung der Fachgruppe Pädagogische Psychologie) (Kiel).

[B3] Cai B. Rajendran K. Bay B. H. Lee J. Yen C. C. (2019). The effects of a functional three-dimensional (3D) printed knee joint simulator in improving anatomical spatial knowledge. Anat. Sci. Educ. 12, 610–618. doi: 10.1002/ase.1847 30536570

[B4] Castro-Alonso J. Hidalgo A. A. Sweller J. (2024). Biological evolution and human cognition are analogous information processing systems. Front. Psychol. 14:1330345. doi: 10.3389/fpsyg.2023.1330345 38250110 PMC10796771

[B5] Chen S. Pan Z. Wu Y. Gu Z. Li M. Liang Z. . (2017). The role of three-dimensional printed models of skull in anatomy education: a randomized controlled trail. Sci. Rep. 7:575. doi: 10.1038/s41598-017-00647-1 28373643 PMC5428829

[B6] Fehrmann R. (2025). Implementing augmented reality models in the classroom environment using merge cubes: a quantitative study of the effects on students' cognitive load and motivation. Educ. Sci. 15:414. doi: 10.3390/educsci15040414

[B7] Fiorella L. (2021). “The embodiment principle in multimedia learning,” in Cambridge University Press eBooks 286–295. doi: 10.1017/9781108894333.030

[B8] Glenberg A. M. (2008). “Toward the integration of bodily states, language, and action,” in Embodied Grounding: Social, Cognitive, Affective and Neuroscientific Approaches, eds. G. R. Semin and E. R. Smith (Cambridge: Cambridge University Press), 43–70.

[B9] Hayes J. C. Kraemer D. J. M. (2017). Grounded understanding of abstract concepts: the case of STEM learning. Cogn. Res. Princ. Implic. 2:7. doi: 10.1186/s41235-016-0046-z 28203635 PMC5281667

[B10] Hidi S. Renninger K. A. (2006). The four-phase model of interest development. Educ. Psychol. 41, 111–127. doi: 10.1207/s15326985ep4102_4

[B11] Hutmacher F. Kuhbandner C. (2018). Long-term memory for haptically explored objects: fidelity, durability, incidental encoding, and cross-modal transfer. Psychol. Sci. 29, 2031–2038. doi: 10.1177/0956797618803644 30376424

[B12] Isen A. M. Reeve J. (2005). The influence of positive affect on intrinsic and extrinsic motivation: facilitating enjoyment of play, responsible work behavior, and self-control. Motiv. Emot. 29, 295–323. doi: 10.1007/s11031-006-9019-8

[B13] Justo E. Delgado A. Llorente-Cejudo C. Aguilar R. Cabero-Almenara J. (2022). The effectiveness of physical and virtual manipulatives on learning and motivation in structural engineering. J. Eng. Educ. 111, 813–851. doi: 10.1002/jee.20482

[B14] Kalyuga S. (2023). Evolutionary perspective on human cognitive architecture in cognitive load theory: a dynamic, emerging principle approach. Educ. Psychol. Rev. 35:1. doi: 10.1007/s10648-023-09812-7

[B15] Kalyuga S. Ayres P. Chandler P. Sweller J. (2003). The expertise reversal effect. Educ. Psychol. 38, 23–31. doi: 10.1207/S15326985EP3801_4

[B16] Keller J. M. (2009). Motivational Design for Learning and Performance: The ARCS Model Approach. New York, NY: Springer.

[B17] Klatzky R. L. Lederman S. J. (2002). “Tactile object perception and the perceptual stream,” in Unfolding Perceptual Continua, ed. L. Albertazzi (Amsterdam: John Benjamins Publishing Company), 147–162.

[B18] Klepsch M. Schmitz F. Seufert T. (2017). Development and validation of two instruments measuring intrinsic, extraneous, and germane cognitive load. Front. Psychol. 8:1997. doi: 10.3389/fpsyg.2017.01997 29201011 PMC5696680

[B19] Kong X. Nie L. Zhang H. Wang Z. Ye Q. Tang L. . (2016). Do 3D printing models improve anatomical teaching about hepatic segments to medical students? A randomized controlled study. World J. Surg. 40, 1969–1976. doi: 10.1007/s00268-016-3541-y 27172803

[B20] Kooloos J. G. Schepens-Franke A. N. Bergman E. M. Donders R. A. Vorstenbosch M. A. (2014). Anatomical knowledge gain through a clay-modeling exercise compared to live and video observations. Anat. Sci. Educ. 7, 420–429. doi: 10.1002/ase.1443 24623632

[B21] Krontiris-Litowitz J. (2003). Using manipulatives to improve learning in the undergraduate neurophysiology curriculum. Adv. Physiol. Educ. 27, 109–119. doi: 10.1152/advan.00042.2002 12928320

[B22] Lespiau F. Tricot A. (2024). Reasoning more efficiently with primary knowledge despite extraneous cognitive load. Evol. Psychol. 22, 1–14. doi: 10.1177/14747049241252694 38840333 PMC11155337

[B23] Luo Z. Dang Y. Xu W. (2019). Academic interest scale for adolescents: development, validation, and measurement invariance with Chinese students. Front. Psychol. 10:2301. doi: 10.3389/fpsyg.2019.02301 31681097 PMC6798182

[B24] Makransky G. Lilleholt L. (2018). A structural equation modeling investigation of the emotional value of immersive virtual reality in education. Educ. Technol. Res. Dev. 66, 1141–1164. doi: 10.1007/s11423-018-9581-2

[B25] Manches A. O'Malley C. (2012). Tangibles for learning: a representational analysis of physical manipulation. Pers. Ubiquitous Comput. 16, 405–419. doi: 10.1007/s00779-011-0406-0

[B26] Manrique M. Mondragón I. F. Flórez-Valencia L. Montoya L. García A. Mera C. A. . (2024). Haptic experience to significantly motivate anatomy learning in medical students. BMC Med. Educ. 24:946. doi: 10.1186/s12909-024-05829-w 39215247 PMC11363654

[B27] Mayer R. E. (2005). “Cognitive theory of multimedia learning,” in The Cambridge Handbook of Multimedia Learning, ed. R. E. Mayer (Cambridge: Cambridge University Press), 31–48.

[B28] Mayer R. E. Moreno R. (2003). Nine ways to reduce cognitive load in multimedia learning. Educ. Psychol. 38, 43–52. doi: 10.1207/S15326985EP3801_6

[B29] McGuinness C. (1990). “Talking about thinking: the role of metacognition in teaching thinking,” in Lines of Thinking, eds. K. Gilhooly, M. Deane, and G. Erdos (New York, NY: Academic Press), 310–312.

[B30] Michel-Acosta P. Pepín-Ubrí J. Chaljub-Hasbún J. (2024). Augmented reality about tropical cyclones in the Dominican Republic: evaluation of learning and cognitive load. J. New Approaches Educ. Res. 13:19. doi: 10.1007/s44322-024-00020-x

[B31] Minogue J. Jones M. G. (2006). Haptics in education: exploring an untapped sensory modality. Rev. Educ. Res. 76, 317–348. doi: 10.3102/00346543076003317

[B32] Moreno R. (2004). Decreasing cognitive load for novice students: effects of explanatory versus corrective feedback in discovery-based multimedia. Instr. Sci. 32, 99–113. doi: 10.1023/B:TRUC.0000021811.66966.1d

[B33] Moreno R. (2006). When worked examples don't work: is cognitive load theory at an impasse? Learn. Instr. 16, 170–181. doi: 10.1016/j.learninstruc.2006.02.006

[B34] Moreno R. Durán R. (2004). Do multiple representations need explanations? The role of verbal guidance and individual differences in multimedia mathematics learning. J. Educ. Psychol. 96, 492–503. doi: 10.1037/0022-0663.96.3.492

[B35] Moreno R. Mayer R. (2007). Interactive multimodal learning environments. Educ. Psychol. Rev. 19, 309–326. doi: 10.1007/s10648-007-9047-2

[B36] Novack M. Goldin-Meadow S. (2015). Learning from gesture: how our hands change our minds. Educ. Psychol. Rev. 27, 405–412. doi: 10.1007/s10648-015-9325-3 26366048 PMC4562024

[B37] Nuszbaum M. Voß A. Klauer K. C. Betsch T. (2012). NFT. Need for Touch Scale – deutsche Fassung [Verfahrensdokumentation und Fragebogen]. Trier: ZPID.

[B38] Paas F. Sweller J. (2012). An evolutionary upgrade of cognitive load theory: using the human motor system and collaboration to support the learning of complex cognitive tasks. Educ. Psychol. Rev. 24, 27–45. doi: 10.1007/s10648-011-9179-2

[B39] Peck J. Childers T. L. (2003). Individual differences in haptic information processing: the “Need for Touch” scale. J. Consum. Res. 30, 430–442. doi: 10.1086/378619

[B40] Pintrich P. R. (2003). “Motivation and classroom learning,” in Handbook of Psychology: Educational Psychology, eds. W. M. Reynolds and G. E. Miller (Hoboken, NJ: John Wiley and Sons), 103–122.

[B41] Ratcliffe J. Tokarchuk L. (2020). “Evidence for embodied cognition in immersive virtual environments using a second language learning environment,” in 2020 IEEE Conference on Games (CoG), 471–478.

[B42] Rau A. Nobis C. Behr A. V. Kesting M. R. (2015). Design of a haptic model for the training of cleft treatment procedures. Simul. Healthc. 10, 128–132. doi: 10.1097/SIH.0000000000000078 25742091

[B43] Reid S. Shapiro L. Louw G. (2018). How haptics and drawing enhance the learning of anatomy. Anat. Sci. Educ. 12, 164–172. doi: 10.1002/ase.1807 30107081

[B44] Sepp S. Howard S. J. Tindall-Ford S. Agostinho S. Paas F. (2019). Cognitive load theory and human movement: towards an integrated model of working memory. Educ. Psychol. Rev. 31, 293–317. doi: 10.1007/s10648-019-09461-9

[B45] Skulmowski A. Xu K. M. (2021). Understanding cognitive load in digital and online learning: a new perspective on extraneous cognitive load. Educ. Psychol. Rev. 34, 171–196. doi: 10.1007/s10648-021-09624-7

[B46] Stadler M. Sailer M. Fischer F. (2020). Knowledge as a formative construct: a good alpha is not always better. New Ideas Psychol. 60:100832. doi: 10.1016/j.newideapsych.2020.100832

[B47] Stull A. T. Gainer M. Padalkar S. Hegarty M. (2016). Promoting representational competence with molecular models in organic chemistry. J. Chem. Educ. 93, 994–1001. doi: 10.1021/acs.jchemed.6b00194

[B48] Sultanova G. (2025). Introducing non-cognitive load to the educational discourse. Front. Psychol. 15:1411102. doi: 10.3389/fpsyg.2024.1411102 39872725 PMC11769952

[B49] Sweller J. (1988). Cognitive load during problem solving: effects on learning. Cogn. Sci. 12, 257–285. doi: 10.1207/s15516709cog1202_4

[B50] Sweller J. (1999). Instructional Design in Technical Areas. Melbourne, VIC: ACER Press.

[B51] Sweller J. (2020). Cognitive load theory and educational technology. Educ. Technol. Res. Dev. 68, 1–16. doi: 10.1007/s11423-019-09701-3

[B52] Tanner J. A. Jethwa B. Jackson J. Bartanuszova M. King T. S. Bhattacharya A. . (2020). A three-dimensional print model of the pterygopalatine fossa significantly enhances the learning experience. Anat. Sci. Educ. 13, 568–580. doi: 10.1002/ase.1942 31904166

[B53] Vandenberg S. G. Kuse A. R. (1978). Mental rotations: a group test of three-dimensional spatial visualization. Percept. Mot. Skills 47, 599–604. doi: 10.2466/pms.1978.47.2.599 724398

[B54] Zitzmann S. Orona G. A. (2025). Why we might still be concerned about low cronbach's alphas in domain-specific knowledge tests. Educ. Psychol. Rev. 37:37. doi: 10.1007/s10648-025-10015-5

[B55] Zou L. Zhang Z. Mavilidi M. Chen Y. Herold F. Ouwehand K. . (2025). The synergy of embodied cognition and cognitive load theory for optimized learning. Nat. Hum. Behav. 9, 877–885. doi: 10.1038/s41562-025-02152-2 40119235

