# Supplementary material for: Feel it to learn it!—Cognitive and motivational effects of haptic learning materials
Source: Front Psychol. 2026 Mar 9;17:1753336. doi: 10.3389/fpsyg.2026.1753336 (PMC13007604; doi:10.3389/fpsyg.2026.1753336)
Supplement: Supplementary file 1 [file Data_Sheet_1.pdf]

Supplementary Material

S1 NFT Items (adaptation of the german version)

Item 1: If I want to learn something new (for myself), I feel the need to be able to touch the possible learning content.
Item 2: I have more confidence that I will be able to understand the learning content correctly if I can touch it while learning.
Item 3: It is easier for me to understand learning content if I can touch it.
Item 4: If I can decide on a learning content, it is important for me to be able to hold it or a plastic image or model of it in my hand.
Item 5: When learning, I feel more confident if I can touch the learning content or a plastic image or model of it beforehand, because I can better understand the content.
Item 6: If I can freely choose a topic to learn about, I mainly look at the possible learning content that I can explore by touching it.
Item 7: In order to find out whether it is worth delving deeper into a learning learning content, you have to have touched it.
Item 8: There is a lot of learning content that I would only understand for sure if I could touch it while learning.
Item 9: When I am learning, I always find myself wanting to touch the learning learning content and want to explore it.

S2 self-conducted prior knowledge test (heart) (adaptation of the german version)

Below are some questions about your prior knowledge of anatomy. Don't worry if you can't answer some of the questions yet.

Item 1: What are the functions of arteries and veins? Tick the two correct statements.

1. Veins carry oxygen-depleted blood back to the heart.
2. Veins carry oxygen-rich blood back to the heart.
3. Arteries carry oxygen-poor blood back to the heart.
4. Arteries carry oxygen-rich blood back to the heart.
5. Veins carry oxygen-depleted blood from the heart to the body.
6. Veins carry oxygen-rich blood from the heart to the body.
7. Arteries carry oxygen-depleted blood from the heart to the body.
8. Arteries carry oxygen-rich blood from the heart to the body.

Item 2: Name all the parts of the heart that you know.

Item 3: How many chambers does the human heart have?
1. 1
2. 2
3. 4
4. 6

Item 4: How is the heart supplied with energy? Select the correct answer.
1. The necessary oxygen is supplied via the pulmonary arteries.
2. Energy comes from the blood flowing in and out of the heart chambers.
3. The heart is responsible for supplying the rest of the body with energy and therefore does not need any energy itself.
4. Coronary arteries around the heart muscle supply the heart with energy.

Item 5: Describe the path of blood in the heart. Where does it flow in and out? How is it enriched with oxygen? Describe your current knowledge of our heart functions as accurately as possible.

S3 self-conducted prior knowledge test (spine) (adaptation of the german version)

Below are some questions about your prior knowledge of anatomy. Don't worry if you can't answer some of the questions yet.

Item 1: What shape does the spine resemble when viewed from the side?
1. I-shaped
2. S-shaped
3. Double S-shaped
4. O-shaped

Item 2: How many sections is the spine divided into, and what are they called? State the number and name all the sections of the spine that you know.

Item 3: What are the components of the spine? Select all applicable answers.
1. Bursae
2. Vertebral bodies
3. Cartilage
4. Intervertebral discs
5. Ribs
6. Capsules
7. Spinal cord
8. Tendons
9. Ligaments

Item 4: How many vertebrae does the human spine have on average?
1. 19
2. 25
3. 33
4. 46

Item 5: What functions does the spine perform in humans?

Item 6: Name all the components of a vertebral body that you are familiar with.

S4 self-conducted learning test (heart) (adaptation of the german version)

Item 1: Which artery distributes blood from the heart throughout the body?
1. Coronary artery
2. Aorta
3. Superior vena cava
4. Pulmonary artery

Item 2: What separates the right atrium from the right ventricle? Select the correct answer.
1. Tricuspid valve
2. Ventriculus dexter
3. Pocket valves
4. Atrium sinistrum

Item 3: Label the illustration.

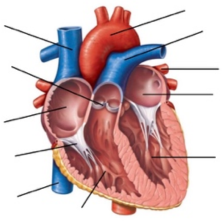

Item 4: Which of the following is NOT a function of the heart?
1. Supplying vital substances to internal organs, muscles and the brain.

2. Re-oxygenating blood that is rich in carbon dioxide.
3. Maintaining blood circulation.

Item 5: Which part of the heart does oxygen-depleted blood pass through first?

Item 6: How can it happen that the heart is not supplied with sufficient energy?

Item 7: Why might it be important for the atria to be separated from the ventricles by heart valves?
1. Regulation of blood flow
2. Prevention of blood backflow
3. There is no known benefit of heart valves
4. Oxygen-poor blood from the atrium is separated from oxygen-rich blood in the ventricle by the heart valve
5. Filtering toxins from the blood
6. Stimulation of the heart's pumping function

Item 8: A patient suffers from gasping for breath, but the doctor cannot find any defect in the lungs and suspects that the cause lies in the heart function. What could be causing the symptoms?
1. Left heart failure: The heart is unable to pump as much blood out of the heart as enters it, causing blood to accumulate in the lungs.
2. Right heart failure: Not as much blood enters the heart as it should, but the blood is quickly pumped into the body. This results in negative pressure in the heart, which affects the lungs.
3. The pulmonary artery appears to be blocked, so that too little blood reaches the lungs to be enriched with oxygen.
4. The pulmonary vein appears to be blocked, so that too little blood reaches the lungs to be enriched with oxygen.
5. The cause of gasping for breath cannot be found in heart function.

Item 9: What can be the consequence of blocked coronary arteries?

S5 self-conducted learning test (spine) (adaptation of the german version)

Item 1: Label the illustration of the spine.

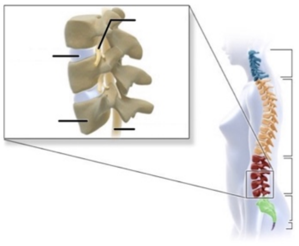

Item 2: Label the components of the vortex.

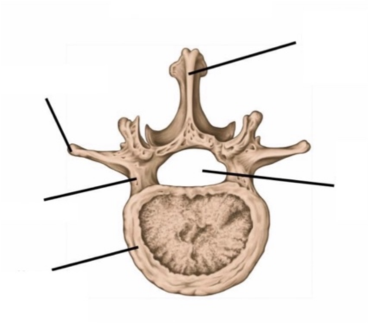

Item 3: Which of the following is NOT a function of our spine?
1. Cushioning our movements
2. Protecting the spinal cord
3. Supporting neighbouring muscle groups
4. Enabling us to walk upright

Item 4: The spine consists of...
1. 15 movable and 19 rigid vertebrae
2. 19 movable and 10-15 rigid vertebrae
3. 24 movable and 9-10 rigid vertebrae
4. 29 movable and 4-5 rigid vertebrae

Item 5: Why are the vertebrae different sizes?

Item 6: The vertebrae are connected to each other by small joints at the articular processes. What is the function of these joints? Select all applicable answers.
1. Prevent excessive rotation to the left and right
2. Cushion movements
3. Secure the intervertebral discs
4. Stabilise the muscles

Item 7: Why do adults become smaller with age?

Item 8: Where is the spinal cord located?

Item 9: What function do the intervertebral discs perform in the spinal column?

Item 10: How many intervertebral discs does a human being have?
1. 17
2. 23
3. 28
4. 33

Item 11: With advancing age, the human spine is threatened by which of the following answers? Tick the appropriate answer.
1. Restricted movement due to the fusion of vertebrae
2. Signs of wear and tear on the vertebrae
3. Signs of wear and tear on the intervertebral discs
4. Decrease in the double S-curve

Item 12: What movements does the spine enable?
1. Forward flexion
2. Backward extension
3. Sideways bending

4. Rotation

Item 13: Why do humans have fewer intervertebral discs than vertebrae?
